# Neural Changes Developed during the Extinction of Cocaine Self-Administration Behavior

**DOI:** 10.3390/ph4101315

**Published:** 2011-10-13

**Authors:** Alejandro Higuera-Matas, Miguel Miguens, Nuria del Olmo, Carmen García-Lecumberri, Emilio Ambrosio

**Affiliations:** 1 Department of Psychobiology, School of Psychology, UNED, C/Juan del Rosal 10, 28040 Madrid, Spain; E-Mail: ahiguera@psi.uned.es (A.H-M.); cglecumberri@psi.uned.es (C.G.L.); 2 Department of Basic Psychology I, School of Psychology, UNED, C/Juan del Rosal 10, 28040 Madrid, Spain; E-Mail: mmiguens@psi.uned.es (M.M.); 3 Department of Pharmaceutical and Nutritional Sciences, School of Pharmacy, San Pablo-CEU University, Urb. Montepríncipe, Boadilla del Monte, Madrid, Spain; E-Mail: nolmo@ceu.es (N.O.)

**Keywords:** cocaine self-administration, extinction, neuroadaptive changes

## Abstract

The high rate of recidivism in cocaine addiction after prolonged periods of abstinence poses a significant problem for the effective treatment of this condition. Moreover, the neurobiological basis of this relapse phenomenon remains poorly understood. In this review, we will discuss the evidence currently available regarding the neurobiological changes during the extinction of cocaine self-administration. Specifically, we will focus on alterations in the dopaminergic, opioidergic, glutamatergic, cholinergic, serotoninergic and CRF systems described in self-administration experiments and extinction studies after chronic cocaine administration. We will also discuss the differences related to contingent versus non-contingent cocaine administration, which highlights the importance of environmental cues on drug effects and extinction. The findings discussed in this review may aid the development of more effective therapeutic approaches to treat cocaine relapse.

## 1. Introduction

While opiate and alcohol addiction may be partially treated with specific pharmacotherapies, no such approach is available for psychostimulant addictions [1]. Moreover, treatment of these disorders is further complicated by a high inherent risk of relapse. These features highlight the need for more effective prevention programs and a better understanding of the neurobiological mode of action of these substances with a view to developing drugs that aid patient recovery. Cocaine acts by blocking voltage-dependent sodium channels (which mediates its analgesic effects) [2] and inhibiting the reuptake of dopamine, serotonin and noradrenaline [3], acting presynaptically at the level of the vesicular monoamine transporter [4] and postsynaptically at M1 and M2 muscarinic receptors [5-7], serotoninergic receptors [8] and sigma opioid receptors [9]. Cocaine also exerts sympathomimetic effects, which appear to be mediated by its noradrenergic activity at the postganglionar terminals of the sympathetic autonomous nervous system. These effects include increased heart rate, mydriasis, vasoconstriction and salivation, gastric and pancreatic secretion. Increased noradrenergic activity in the *locus coeruleus* also mediates the increase in alertness and arterial pressure [2,10-12].

A growing body of evidence suggests that the dopaminergic effects of cocaine are responsible for its psychomotor, rewarding and euphoric effects [13-16]. There is some controversy with regard to this issue as the reinforcing potency of cocaine was reported to be maintained in the absence of the DAT but decreased in the absence of the NET and its motivational rewarding effect was observed in the absence of the SERT, but not when both DAT and SERT are lacking [17]. However, impaired cocaine self-administration was more recently demonstrated in mice lacking the dopamine transporter [18].

From a neuroanatomic point of view, most of cocaine's rewarding and psychomotor activities appear to be dependent upon the integrity of the mesocorticolimbic dopaminergic system. Chronic cocaine intake is associated with functional alterations in specific neuronal populations within this circuit, as well as specific modulatory effects on neurotransmitter receptors, molecular signalling cascades and gene expression [19]. While much research effort in the last three decades has focused on cocaine's effects on the dopaminergic system, it should not be forgotten that this drug also affects the serotonin, noradrenaline, opioid, glutamate, GABA and corticotropin releasing factor (CRF) neurotransmitter systems (see below).

## 2. Mechanisms Involved in Cocaine Relapse

Relapse is one of the greatest barriers to the effective treatment of drug addiction. Craving and compulsive use of a drug are two central features of this phenomenon [20-22]. Two opposing theories have emerged to explain relapse. The first suggests that relapse occurs after activation of reward circuits, as observed following acute drug administration, which supports the existence of a proponent process [23,24]. An alternative hypothesis proposes the existence of an opponent process elicited by drug administration, which induces hypofunctionality in reward circuits that is ultimately translated to dysphoric states during drug withdrawal [25-28]. However, both theories fail to fully account for several relapse-related phenomena. For example, the opponent process theory is contradicted by the following observations: (1) periods of maximal drug self-administration do not always overlap with periods of maximal dysphoria, *i.e.*, for a wide variety of drugs of abuse there is a poor correlation between drug withdrawal effects *per se* and drug craving [29]; (2) there is a large body of medical and experimental evidence indicating that the relief of withdrawal symptoms is not an effective method to treat addiction [23]; (3) passive drug administration to animals in which drug seeking has been extinguished reinstates this behavior [30]. These data suggest the existence of a drug craving mechanism independent of a negative reinforcement process. Taken together, this evidence suggests that the two core features of addiction (drug craving and relapse) are unrelated to the desire to escape the aversive consequences of abstinence.

The proponent process theory proposes a positive reinforcement process, yet it is similarly contradicted by several findings suggesting that positive reinforcement and hedonic states are not correlated. In the first place, several psychostimulant drugs that do not produce euphoria are still highly addictive, such as nicotine [31]. Moreover, drug intake is maintained despite the dysphoric states that may be associated with the initial phases of drug use and the negative consequences associated with long term use [23]. In addition, the intense pleasure that a drug may produce when administered acutely does not appear to be derived when the individual is exposed to environmental stimuli previously associated with the drug, although these stimuli can induce a robust relapse to drug seeking [30]. These observations point to a dissociation between the pleasure induced by acute drug administration and the desire to consume the drug. Finally, humans self-administer low doses of drugs that in themselves do not cause pleasure, as witnessed with cocaine and opiates [32]. Based on these and other findings, it is clear that neither of the two theories can fully explain the phenomenon of relapse. However, both theories suggest that drug abuse produces specific neural adaptations that mediate the intense craving and drug seeking behavior observed long after chronic drug intake. Chronic drug administration may induce two types of neuroadaptive changes. One such change occurs firstly as a direct consequence to the pharmacological effects of the drug and it may generate tolerance to the physiological effects of the drug. The second type of adaptation is derived from the strong associations between the reinforcing effects of the drug and the environmental stimuli associated with them. Hence, stimuli that are motivationally neutral may acquire the ability to elicit the same responses as the drug itself [33]. Considering the importance of such neural adaptations in drug craving and relapse, we will summarize the key findings of our group and other relating to the neural adaptations that occur during the extinction of cocaine self-administration.

## 3. Neural Changes Induced after the Extinction of Cocaine Self-Administration Behavior

The main experimental design we use in our studies involves a triad approach employing three groups of animals. The first group of animals can press a lever to obtain a drug infusion and thus, they can exercise contingent control on their self-administration behavior. The second group is yoked to the first and as such, the animals passively receive the drug whenever the rats of the first groups press a lever for an infusion. This group is used to control for the effects of contingency. The third group receives non-contingent saline injections and serves as a control for the pharmacological effects of the drug [34,35]. Using this basic design, we have analyzed the neural adaptations that occur after cocaine administration and during extinction (1, 5 and 10 days), when cocaine is substituted with a saline solution.

### 3.1. The Dopaminergic System

Using the design described above, we measured the levels of D1 and D2 dopamine receptors by quantitative autoradiography. The levels of D2-like dopamine receptors decrease in several forebrain regions after cocaine self-administration, an effect that is maintained throughout the extinction period. Interestingly, the long-term down-regulation of D2 receptors in cocaine-treated animals is more evident in self-administering rats than in yoked animals. Moreover, neither contingent access to cocaine nor passive administration of the drug affects dopamine D1 receptors in these experiments. These results are in agreement with previous reports in the literature. For example, chronic cocaine self-administration in monkeys leads to decreased binding level of D2 receptors in the anterior and central regions of the caudate nucleus, putamen, olfactory tubercle, and both the shell and core of the nucleus accumbens (NAcc) [36]. In the rat brain, a decrease in the binding levels to D2 receptor sites was also observed after withdrawal from limited but not extended access to cocaine, while D1 receptors were transiently up-regulated following 20 minutes of withdrawal [37].

Analysis of dopamine transporter (DAT) binding in these animals reveals an increase in DAT binding in the caudate-putamen (CPU), NAcc (core and shell) and ventral tegmental area (VTA) in response to contingent cocaine administration. Furthermore, when compared with saline and cocaine-yoked animals, this increase in binding was maintained during the entire extinction period in most of the brain areas examined. These results are in agreement with previous findings that showed that the DAT up-regulation evident after cocaine self-administration endured after withdrawal [38]. There are, however, mixed results in the literature concerning the regulation of DATs after cocaine exposure and withdrawal. For example it has been shown that cocaine self-administration may lead to either decreases or increases in the number of DATs as a function of the self-administration phase. For example, the Porrino group showed that the DAT levels decreased after initial exposure to cocaine but increased with higher doses and after several self-administration sessions, moreover, these changes were region-specific [39]. In contrast, limited access to cocaine was shown to up-regulate DAT levels in another study, while extended access was ineffective in provoking such changes [40]. The reasons for the discrepancies may lay on the different species used in both studies (rhesus monkeys *vs.*, rats), or the specific self-administration parameters used (number of sessions or session duration, for example). Other authors have found a significant up-regulation of the DAT following extended access and withdrawal, but these changes were restricted to the prefrontal cortex [20].

With regard to human cases, there are at least 10 *postmortem* studies that employed either *in vitro* binding or autoradiography techniques to evaluate and contrast the status of striatal DAT in cocaine dependence with matched healthy controls. The results of the studies are not consistent, with reports of significant increases, decreases, and no change in DAT in cocaine dependent subjects relative to controls (see [41] for a review).

It has been reported that DAT binding augmented and the expression of dopamine D2 receptors diminished after long-term chronic cocaine self-administration in monkeys [36,39,42]. Accordingly, we have found a decrease in D2 receptor binding that persisted even 10 days after the extinction [43].The coupling of increased DAT protein binding with a decrease in D2 receptor binding suggests an enhancement of dopamine transmission. In the absence of cocaine, increased DAT activity would lead to enhanced clearance of synaptic dopamine, thereby dampening dopamine neurotransmission. However, no changes in basal extracellular dopamine levels have been reported in the NAcc after long term extinction of cocaine self-administration [44].

Chronic cocaine self-administration also decreased tyrosine hydroxylase (TH-the rate-limiting enzyme for dopamine synthesis) protein levels in the NAcc shell (but not core) after one week of withdrawal from self-administration [45]. In contrast, repeated extinction training during a one week withdrawal period completely normalized deficits in TH to levels found in untreated controls [45].This same group reported that TH levels in VTA dopamine neurons were not altered after one week of withdrawal from chronic cocaine self-administration [45]. However, extinction training during withdrawal increased TH in the VTA over control levels. Thus, in both VTA and NAcc, extinction-induced regulation represents an increase in TH relative to animals control animals without extinction training. These results suggest that normalization of TH deficits in the NAcc could result from an increased TH synthesis in the VTA leading to greater transport to dopamine terminals in the NAcc. Alternatively, extinction training could stabilize or impair degradation of TH in dopaminergic terminals of the NAcc shell; see [46] for an interesting review on this issue.

### 3.2. The Glutamatergic System

In addition to the changes reported in the dopaminergic system, NMDA glutamate receptor expression is altered 1 day after the extinction of cocaine self-administration. This change is short-lived and no such changes are observed 5 or 10 days after extinction. Furthermore, this cocaine-induced modulation is dependent upon the mode of cocaine administration and while NMDA receptors augment in animals that self-administer cocaine, they decrease in those receiving yoked infusions (unpublished observations). When expression of the NMDAR1 subunit of the NMDA receptor was assessed, it increases in forebrain regions involved in the cocaine-reinforcing effects of the non-contingent group during cocaine administration and subsequent extinction. Similar alterations in NMDAR1 mRNA are observed in all the brain areas analyzed, although the magnitude of the changes vary in each brain region and in function of the mode of cocaine administration (contingent versus non-contingent). Across the brain, NMDAR1 gene expression is upregulated by contingent cocaine administration on the last day of drug intake when compared with saline or non-contingent cocaine administration. In the absence of cocaine in the contingent group, NMDAR1 mRNA expression decreases progressively, an effect that persists for up to 10 days after extinction in all forebrain areas except the olfactory tubercle (TU). By contrast, non-contingent cocaine administration does not change NMDAR1 gene expression on the last session of cocaine intake, while drug withdrawal in this group increases the NMDAR1 mRNA transcripts on Days 1 and 5 of extinction. However, this increase returns to the basal (saline) level 10 days after the last non-contingent drug administration session. These results suggest that interaction between environmental stimuli and the pharmacological action of cocaine during self-administration and extinction is important for cocaine-mediated regulation of NMDAR1 gene expression. Furthermore, the sustained decrease in NMDAR1 mRNA 10 days after extinction in the contingent cocaine group is not consistent with a response to short-term compensatory adaptations in brain function in the forebrain region [47].

Other groups have found that extinction training increases the amount of GluR1 and GluR2/3 subunits of AMPA glutamate receptors in the NAcc shell subregion [48]. In contrast to GluR1 and GluR2/3, the NR1 subunit of NMDA receptors decreased in the NAcc shell after one week of withdrawal from chronic cocaine self-administration. However, extinction training prevented deficits in NR1. This effect also required access to the response levers, and hence, extinction of cocaine-seeking instrumental responses. These results are opposed to those previously reported by us (see above) and several methodological reasons, such the number of self-adminstration or extinction sessions, or the technique used to measure NR1 levels (Western blot *vs. in situ* hybridization) may account for these discrepancies. The extinction-induced changes in GluR2/3 and GluR1 content have functional consequences on extinction behavior and subsequent relapse. In this sense, it has been shown that viral-mediated GluR2 and GluR1 overexpression in the NAcc shell facilitates extinction and attenuates reinstatement [48].

Extinction training after cocaine self-administration has also been shown to induce glutamatergic plasticity to inhibit cocaine seeking [49]. In this study, rats were either extinguished or withdrawn without extinction training from cocaine self-administration, and measurements of postsynaptic density proteins in the core and shell subcompartments of the NAcc were compared with yoked-saline controls. Only extinguished rats had elevations of PSD-95, Homer1b/c, and Narp in the postsynaptic density of the NAcc core, with no changes in the shell. The authors also reported that surface expression of mGluR5 was reduced only in the core of extinguished animals, suggesting that that the elevation in Homer1b/c in the core may have sequestered mGluR5 away from the membrane surface and that the loss of surface mGluR5 could have inhibited long-term depression. This could be a cellular mechanism that may contribute to the inhibition of cocaine seeking after extinction of the self-administration behavior [49].

Glutamate levels are controlled by the excitatory aminoacid transporters (EAATs). We observed changes in glutamate transporters after cocaine self-administration and subsequent extinction. We detected a decrease in binding to EAATs, the family of glutamate transporters, in the CA1 subfield of the hippocampus and the cerebellar cortex after chronic cocaine self-administration. By contrast, binding to EAATs increases after extinction of this behavior for 1 day, but only in the infra-limbic portion of the medial prefrontal cortex. No other differences in EAAT binding levels were observed in any of the brain regions analyzed. However, it should be noted that the radioligand used in this study could not differentiate between the different subtypes of EAATs involved in glutamate transport and thus, changes in the expression of a particular EAAT subtype could be masked by the expression of another subtype. Interestingly, the neuroadaptive changes in EAAT binding were only detected in animals receiving contingent cocaine administration, in accordance with the differential neurochemical effects in response to contingent versus non-contingent cocaine administration described elsewhere [47,50,51]. Notably, these changes in EAAT binding were reversible, as they were no longer detected after 5 or 10 days of extinction.

In another study we set out to define the time course of the effects of cocaine self-administration and extinction on glutamate and aspartate levels in the NAcc. Rats were trained to self-administer cocaine for 20 days, and the levels of extracellular glutamate and aspartate measured by *in vivo* microdialysis during cocaine self-administration and after a priming cocaine injection at different time points after extinction (1, 5, or 10 days). A food-reinforced control group was also included in the study. In addition, we evaluated the effect of acute i.v. cocaine administration (0, 1, 2, or 4 mg/kg) on glutamate and aspartate levels. We found that at all doses tested, acute i.v. cocaine has no effect on the levels of glutamate or aspartate in the NAcc. By contrast, glutamate levels are reduced in animals trained to self-administer cocaine, although substantial increases are evident during a subsequent session of cocaine self-administration and no such effects are observed in food-reinforced controls. After 1 or 5, but not 10 days of extinction, glutamate levels are reduced. The ability of i.v. cocaine priming injections to increase glutamate levels followed a similar time course, and these effects were specific, as aspartate levels are not affected by any administration protocol. Taken together, these results suggest that recent chronic cocaine administration is necessary to reduce basal glutamate in the core of the NAcc, and that glutamatergic transmission is involved in both the maintenance of cocaine self-administration and the early phases of abstinence [52].

Glutamatergic and dopaminergic transmission are intimately associated with plastic processes such as long-term potentiation (LTP) [53,54]. As such, we analyzed the status of LTP in the hippocampus of rats after extinction of cocaine self-administration. In hippocampal slices from animals in which chronic cocaine self-administration behavior was extinguished, high frequency stimulation evokes greater LTP than that observed in animals that self-administered saline. Thus, cocaine self-administration appears to induce long-lasting changes in hippocampal synaptic plasticity that are maintained 10 days after the last self-administration session. While the mechanisms involved in this facilitation of LTP remain unknown, it is tempting to speculate that chronic self-administration of cocaine induces enduring metaplasticity in the hippocampus, facilitating neuronal responses to stimuli such as a single tetanus spike. In addition, our data suggest that neuroadaptive changes in hippocampal synaptic transmission play an important role in the long-term addictive potential of cocaine [55].

### 3.3. The Opioidergic System

To analyze the endogenous opioid system, we studied the effect of long-term self-administration of cocaine and its subsequent extinction on PENK gene expression in the CPu, NAcc, Tu, piriform cortex (Pir), central nucleus of amygdala (Ce) and ventromedial hypothalamic nucleus (VMN). The magnitude and significance of the changes in PENK gene expression vary, depending on the brain region examined and the mode of drug administration (contingent versus non-contingent). Both contingent and non-contingent cocaine administration increase the expression of PENK on either the last day of cocaine self-administration or during extinction in all the brain regions examined, with the exception of the VMN and Ce nuclei where significant decreases were detected in contingent animals when compared to non-contingent and control animals. In the VMN, the decrease in PENK gene expression returns to basal levels 10 days after the extinction of cocaine self-administration behavior, while this decrease is maintained in the Ce for the entire 10-day period of extinction. These sustained effects on PENK gene expression do not suggest a response to short-term compensatory adaptations in brain function after cessation of cocaine self-administration. Interestingly, our results reveal important differences in PENK gene expression in some brain regions that depend upon the mode of cocaine administration. Although PENK mRNA expression differs in most brain regions of the animals that received cocaine, with the exception of VMN, the changes persisted mainly in the cocaine contingent administration group 10 days after extinction. These results suggest that the interaction between environmental stimuli and the pharmacological action of cocaine during drug self-administration and extinction may represent an important variable in cocaine-mediated regulation of PENK gene expression [51] which could also have important implications for the regulation of relapse into cocaine seeking [56].

### 3.4. The CRF System

Given the growing importance of CRF transmission in addiction [26,57], we considered it essential to analyze CRF expression in our experimental model [50]. Accordingly, we demonstrated that CRF gene expression is altered in the paraventricular nucleus (PVN) of the hypothalamus, whereby long-term cocaine self-administration and extinction provoke a large concentration of neurosecretory CRF cells. As observed for other neurotransmitter systems, the mode of cocaine administration appears to be an important variable in regulating CRF gene expression in the PVN. While long-term cocaine self-administration has no effect on CRF mRNA expression in the PVN, passive cocaine administration in yoked subjects dramatically reduces CRF expression. In our experimental design, animals that self-administer cocaine can regulate and control their own cocaine intake, whereas the yoked cocaine administered animals have no such control over their intake. Thus, these results suggest that environmental events associated with cocaine self-administration may play an important role in mediating the effects of cocaine on hypothalamic CRF mRNA expression.

### 3.5. The Cholinergic System

As stated in the introduction, cocaine acts postsynaptically at the level of M1 and M2 muscarinic receptors and therefore, neural adaptation of these receptors may occur during cocaine self-administration and extinction. Indeed, muscarinic cholinergic receptors modulate dopaminergic function in brain pathways thought to mediate cocaine's abuse-related effects. Consequently, an attenuation of cocaine's reinforcing and discriminative stimulus effects via muscarinic M1 acetylcholine receptor stimulation has been recently reported [58]. Additionally, M2 receptors in the lateral dorsal tegmental area modulate cocaine reward as M2 agonist infusions in the lateral dorsal tegmental area reduced cocaine self-administration [6]. In addition, activation of muscarinic and nicotinic acetylcholine receptors in the NAcc-core is necessary for the acquisition of drug reinforcement [59]. When we analyzed the levels of M1 and M2 receptors in the three groups of animals used in our experimental paradigm, contingent cocaine administration augments the muscarinic receptors in the forebrain (cingulate cortex and other regions). Interestingly, these changes were not evident in other caudal regions, such as the VTA and the caudal mesencephalon, although non-contingent cocaine administration augments the muscarinic receptors found in these regions, as well as in the NAcc. In limbic areas such as the hippocampus, both contingent and non-contingent cocaine administration decrease the levels of muscarinic receptors. These changes remain stable throughout the extinction period, highlighting the enduring nature of neural adaptations in the cholinergic system induced by cocaine self-administration [60]. These results are consistent with the fact that cocaine priming-induced reinstatement is mediated, in part, by increased signaling through muscarinic acetylcholine receptors in the shell subregion of the NAcc [61]. The literature regarding the role of muscarnic receptors on human cocaine addiction is rather scarce. There is one report providing evidence for altered neural cholinergic receptor systems in cocaine-addicted subjects. In this work, it was shown that cocaine addicted subjects and controls differ in their subcortical, limbic, and cortical response to cholinergic probes in areas relevant to craving, learning, and memory [62]. Therefore, it can be suggested that cholinergic systems may offer a pharmacologic target for cocaine addiction treatment.

## 4. Conclusions

The data presented above suggest that cocaine self-administration modifies a number of neurotransmitter systems that might influence susceptibility to relapse. In particular, a divergence can be seen in the effects of contingent versus non-contingent exposure to cocaine, which serves to highlight the importance of goal-directed behavior in mediating the neuromodulatory effects of cocaine. Another important observation is the persistence of the neuroadaptive changes in regulatory elements of the dopaminergic, opioidergic and cholinergic systems during the extinction period. Hence, long term adaptations in these neurotransmitter systems play an important role in the relapse phenomenon, representing potential pharmacotherapeutic targets to deal with cocaine addiction.
